# The selection performance of an antibody library displayed on filamentous phage coat proteins p9, p3 and truncated p3

**DOI:** 10.1186/1756-0500-7-661

**Published:** 2014-09-19

**Authors:** Tuomas Huovinen, Markku Syrjänpää, Hanna Sanmark, Titta Seppä, Sultana Akter, Liton Md Ferdhos Khan, Urpo Lamminmäki

**Affiliations:** Department of Biochemistry, University of Turku, Turku, 20520 Finland

**Keywords:** Phage display, Hyperphage, VCS-M13, Fab, ScFv, g3p, g9p, p3, p9

## Abstract

**Background:**

Filamentous phage display has become an ordinary tool to engineer antibody fragments. Several capsid proteins have been applied for displaying antibodies, of which gene III (p3) protein is used the most followed by experiments with gene IX (p9) protein. Despite the popularity, there are no library scale studies to objectively compare differences in the selection performance of the libraries, when displayed via different capsid proteins.

**Results:**

In this study, an identical antibody repertoire was displayed as Fab fragments on p9, p3 and truncated p3 (p3Δ). In addition, the library clones were displayed as ScFv fragments on p3Δ and the Fab-p3 display valency was modulated by hyperphage and VCS-M13 superinfections. The selection performances of the libraries were followed in repeated parallel panning reactions against streptavidin (STR) and digoxigenin (DIG). Selection was successful with all display formats, but the enrichment of specific clones from Fab-p9 library was clearly less efficient than from the other libraries. The most diverse outputs were obtained from p3Δ display and the highest affinity anti-DIG antibodies from the ScFv repertoire. Unfortunately, the number of retrieved specific clones was too low for explicit analysis of the differences in the number of obtained unique clones from each library. However, severe reduction in sequence diversity was observed in p3-Fab libraries prior to panning, which in turn, materialized as a low number of unique specific clones. Oligovalent display by hyperphage resulted in a higher number of unique clones, but the same highest affinity anti-DIG Fab was recovered also by VCS-M13 superinfection.

**Conclusions:**

The compromised enrichment of the target-specific clones from the Fab repertoire as a fusion to p9 capsid protein in our experiments, the significant loss of functional diversity in Fab-p3 library after single phage packing cycle and the retrieval of higher affinity anti-digoxigenin clones as ScFv molecules than as Fab molecules from the same source repertoire indicate that the chosen display format may have a significant impact on the selection outcome. This study demonstrates that in addition to library content, also display related issues, should be taken into consideration when planning directed evolution experiments.

## Background

A timeline analysis of the number of publications mentioning either “hybridoma” or “phage display” (in title or abstract, accessed via PubMed/ MEDLINE) manifests the change that has happened in the research of bioaffinity reagents, of which, antibodies are still the most used. According to the timeline analysis, hybridoma technology was intensively researched in the 80s and early 90s, but since year 2000 phage display has been cited more often in scientific articles. Naturally, hybridoma technology has become an ordinary platform, which is still the most central source of antibodies for every day work, although not particularly mentioned. On the other hand, phage display is used also for other than antibody engineering efforts. Still, a review of 100 accessed original research papers, covering 1/4 of all “phage display”-citing articles published in year 2009 (Sep–Dec), confirms that antibody fragments were displayed in 50% of the studies, followed by peptides with a 30% share. The remaining 20% of studies concerned display of alternative scaffolds or translated cDNA/gDNA libraries. Sorting the same sample pile of 100 articles by phage type demonstrated that filamentous phage is still by far the most popular choice for *in vitro* evolution studies (91/100) followed by T7 phage display (4/100).

The popularity of using filamentous phage for antibody display stems probably from the compatibility of the phage life cycle with folding of antibody fragments, i.e. Fabs, ScFvs and sdAbs, in the oxidizing environment of the periplasmic space [1]. The fragments are 1/3 to 1/12 size of the full-length antibody and thus, far easier to manipulate by genetic engineering than the full-length genes. Detailed protocols are available both for naïve and synthetic antibody library construction [2, 3], and establishment of a phage display library technology is at the reach of any company or academic institution with a fairly low cost. At present, phage displayed antibody libraries rival traditional hybridoma strategy as a faster, better automatable and more cost-effective route to access monoclonal antibodies. Synthetic antibody repertoires can be even predesigned to exclude unwanted sequence motifs, such as mammalian glycosylation sites, and to limit the library to framework combinations that are well-known for their high expression and aggregation-resistance [4].

Several coat proteins of filamentous phage have been used for antibody display, but a review of the sample group of articles confirmed that the gene 3 protein (p3) is dominating the field with single reports of using gene 9, 7 and 8 proteins (p9, p7 and p8) [5, 6]. In the past, the effect of the signal peptides on the display of Ab fragments [7, 8], the arrangement of the immunoglobulin domain genes on the phagemid [9] and the choice of helper phage used for rescue [10] were studied in detail, but there are no library scale studies concentrating on the effect of the fusion partner to the antibody library display and performance. Comparison studies are, however, crucial information for all phage display users as display method itself may have a major impact on the library function [11, 12]. This aspect should be taken into consideration, when interpreting the selection outcome. In this study, identical Fab library diversity was displayed on the coat proteins p3, truncated p3 (p3Δ, the C-terminal domain of p3 lacking infection domains N1 and N2) and p9. The Fab display efficiency of the libraries and the quality of the displayed repertoires were studied in detail followed by repeated panning experiments to monitor their performance in selections. In addition, the same VL/VH repertoire was selected in parallel in scFv-p3∆ format and the effect of valency modulation by two helper phage, VCS-M13 and hyperphage, was examined.

## Results and discussion

### Library construction

**Figure 1:**
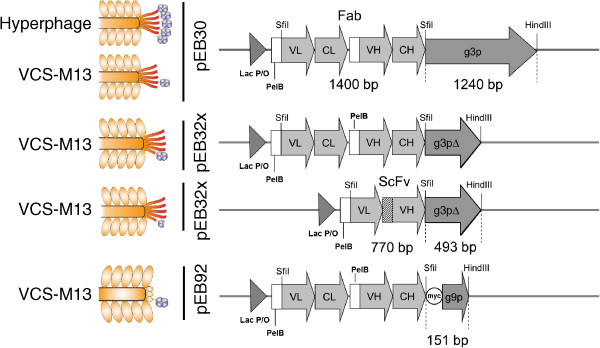
**Phage display formats and genetic constructions.**

Fab libraries were constructed on a template sequence, FabM_GUG_, which was described in Huovinen et al. [13]. FabM_GUG_ is a chimer of mouse constant domains and human variable domains (IGKV3-20 and IGHV3-23), with a display improving mutation M_GUG_, which changed the start codon of the PelB signal sequence of the PelB-VH-CH1 chain from ATG to GTG. The VL and VH variants for the Fab libraries originated from the beta-lactamase-purified light (LC-BLA) and heavy chain repertoires (Fd-BLA) as described for ScFvM construction [14]. In the antibody library, two, six, two and four positions were randomized in the CDR-L1, CDR-L3, CDR-H1 and CDR-H2 loops, respectively, with the majority of diversity introduced at the CDR-H3 loop containing variating loop lengths from 5 to 12 residues (IMGT definition). Same amount of randomized SfiI-digested Fab gene cassette was inserted into the phagemids pEB30, pEB32x and pEB92 containing the full-length p3, truncated p3 (p3∆) and p9 genes, respectively. The ScFv repertoire was identical to the ScFvM library described in Huovinen et al. [14]. The display constructions and expected phenotypes on phage are depicted in Figure 1. The detailed linker sequence between the antibody fragment and the capsid protein was AASGA-EKDIR in full-length p3 display, AASGA-EKIEGR in p3Δ display and AASGA-myc-GGSGG in p9 display. AASGA (or ASGA in ScFv construct) is extra sequence due to the integrated SfiI site and EKDIR and EKIEGR were added for promoting trypsin elution. Also the myc-tag contains a lysine, which sensitizes the p9-Fab construction to trypsin cleavage at this region.

Colony counts of the transformed libraries varied from 1×10^9^ to 6×10^9^ cfu per library (Table 1). Quality of the Fab genes was analysed by sequencing 24 single clones from pEB30-Fab library transformation, of which 20/24 clones contained changes according to the library design and 2/24 clones contained frameshifts (Table 1). The remaining two clones contained a CDR-H3 loop shorter or longer than designed. Similar share of functional clones was anticipated from the other libraries prior to phage packing, as the same Fab cassette pool was used in ligations and the Lac P/O-driven expression of the fusion gene products was continuously suppressed with glucose.

**Table 1:** **Library size, quality and phage titer**

Phagemid	Fusion protein	Transformed	Superinfection	In-frame	Phage yield ^a^I, cfu	Phage yield ^a^II, cfu
pAK400-Fab	LC-BLA	2 × 10^7^	None	8/8	-	-
pEB07-Fab	Fd-BLA	3 × 10^8^	None	8/8	-	-
pEB30-Fab	Fd-p3	3 × 10^9^	None	22/24	-	-
pEB30-Fab	Fd-p3	-“-	Hyperphage	7/23	1.8 ± 0.3 × 10^12^	2.9 ± 1.0 × 10^11^
pEB30-Fab	Fd-p3	-“-	VCS-M13	8/22	7.1 ± 0.5 × 10^13^	3.6 ± 0.8 × 10^12^
pEB32x-Fab	Fd-p3Δ	1 × 10^9^	VCS-M13	22/24	1.5 ± 0.6 × 10^14^	1.1 ± 0.1 × 10^14^
pEB92-Fab	Fd-p9	5 × 10^9^	VCS-M13	22/23	3.3 ± 0.2 × 10^14^	2.9 ± 0.8 × 10^14^
pEB32x-scFv	Fd-p3Δ	6 × 10^9^	VCS-M13	21/34	3.2 ± 0.3 × 10^14^	3.1 ± 7.4 × 10^14^

^a^Phage yield of the display stocks as infective titer per 500 ml cell culture. Titer was measured twice independently (I and II) of three sample dilution series for each sample. Uncertainty informed as the standard deviation of the replica dilutions.

The libraries were superinfected with VCS-M13 for monovalent display, and the Fab-p3 repertoire also with hyperphage for oligovalent display. Hyperphage is a helper phage, which does not provide any wild type p3 for particle assembly and therefore, all p3 originates from the phagemid leading to forced oligovalent display of the protein of interest [15]. Consequently, hyperphage was not used for the superinfection of the p9 or truncated p3 display libraries as this would result in non-infective phage. After the first round of panning with the Fab-p3 oligovalent library, the cells were infected with hyperphage and VCS-M13 in parallel. By the latter strategy, the possible benefits provided by the oligovalent display format on the first round were coupled to the increased selection stringency provided by the monovalent display on following rounds. A library propagated by this manner is referred to as hyper > VCS in continuation.

After phage production, a sample of each library was infected to XL-1 Blue and plated on selective agar plates for picking and growing colonies for sequencing. The single phage-packing cycle decreased the proportion of variants with open reading frames (ORF) in the pEB30-Fab libraries from the previously observed 92% to 36% (VCS-M13) and 30% (hyperphage) depending on the helper phage used in the superinfection. The least influenced libraries in this respect were pEB32x-Fab and pEB92-Fab with 92% and 100% of sequenced clones in ORF after VCS-M13 passage. Surprisingly, only 62% of the pEB32x-ScFv library clones were in ORF, although ScFv in general is considered as the easier display format [16]. The frameshift frequency in pEB30-Fab and pEB32x-scFv library differed significantly from the frequency observed prior to phage packing by *χ*2 test (p < 0.05).

Two out of 34 sequenced ScFv clones contained mutations only in the linker region that had arisen in the last joining step of the variable domains with the repetitive Gly-Ser-linker. Naturally, these mutations are absent in Fab libraries. The frameshift-causing mutations in all libraries were exclusively at the hybridization sites of the mutagenesis primers with majority around the light chain complementary determining regions (CDRs).

### Medium optimization for display

The relative phage titers and display properties of Fab libraries were experimented by exposing cells to various induction conditions in phage production phase. Dilutions of the overnight cultured medium supernatant containing Fab-displaying phage were applied on anti-mouse IgG and anti-phage microtiter plates. The bound phage were revealed with europium-labeled anti-phage antibody. The ScFv-library was left out from this analysis, as the employed capture reagent, rabbit anti-mouse IgG antibodies (RAM), does not bind human ScFv.

**Figure 2:**
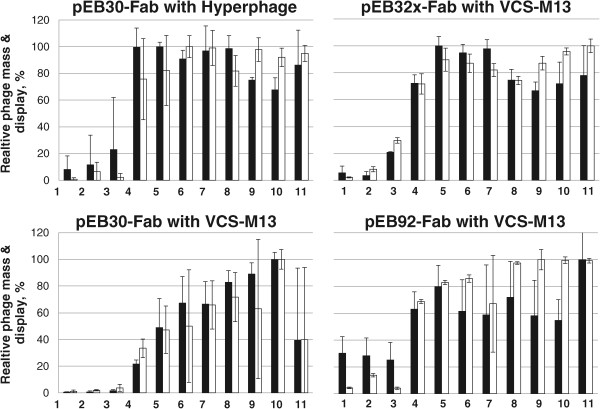
**The effect of LacP/O regulation on relative phage titer and Fab display.** The infected cells were exposed to (1) 1% glucose, (2) 0.5% glucose and 500 μM IPTG, (3) 0.5% glucose, (4) 0.2% glucose, (5) 0.1% glucose, (6) 0.05% glucose, (7) 0.025% glucose, (8) no supplement, (9) 10 μM IPTG, (10) 100 μM IPTG and (11) 250 μM IPTG. Black bar: phage and white bar: Fab. The results are based on three independent experiments with standard deviation shown.

**Figure 3:**
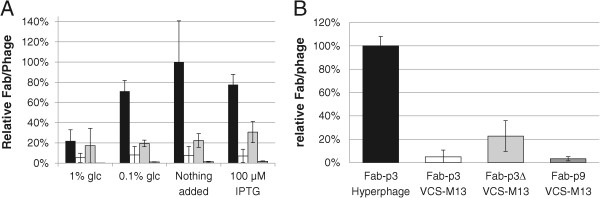
**Influence of the phagemid and helper phage on display level as normalized A) by total phage mass and B) by infectivity.** Black: hyperphage/Fab-p3, white: VCS-M13/Fab-p3, light grey: VCS-M13/Fab-p3Δ and dark grey: VCS-M13/Fab-p9. The data is plotted as the mean of three independent experiments in **A** and as the mean of three replicas in **B** with standard deviation.

The phage titer and glucose concentration were inversely correlated with all libraries, although this phenomenon was only expected with hyperphage-packed Fab-p3 (Figure 2). At high glucose concentrations of 1–0.5% the relative phage titer was only 1.6–25% of the maximum titer reached at lower glucose levels. Reason for this remains unknown. A similar phenomenon was observed in a glucose titration experiment by Kirsch et al. [9] with a single Fab clone displayed with hyperphage from a monocistronic phagemid [9]. This was however not observed with any other display vector in their study, including a bicistronic vector with a soluble light chain and Fd-p3 fusion which is closest to our format. Kirsch et al. used TOP10F’ and we XL-1 Blue as the host strain, which may explain some of the apparent discrepancy in the results. On the other hand, in that experiment the highest sampled glucose concentration was only 0.36% w/v [9], which is necessarily not enough to cause full reduction of titers as observed with Fab-p3∆ library in our experiments (Figure 2).There was a significant, but not dramatic, increase in the display efficiency in hyperphage-infected Fab-p3 and VCSM13-infected Fab-p9 libraries, when moving from Lac P/O repression (glucose) to induction (IPTG) as measured by Fab/phage-ratio (Figure 3A). In general, all media with less than 0.2% glucose seemed to provide the maximum phage titer and display. The natural variation in phage production and display between repeats hindered us from deducing further conclusions.

In some phage library studies 100 – 250 μM IPTG has been used to induce the display of the proteins of interest [17, 18], but the most common practice seems to be abstaining from IPTG induction in the display phase [2, 19, 20]. According to our results, both practices are applicable. However, 250 μM IPTG should be avoided as it does not provide any further benefit. Furthermore, the overexpression of the fusion protein is a significant risk factor promoting the enrichment of aberrant gene clones [21]. One interesting induction option by Krebber et al. [22] was to add both 1% glucose and 500 μM IPTG [22], but at least we could not observe any advantage from this combination in our experiment (Figure 2).

### Display levels

In contrast to the minor impact of Lac P/O regulation on the display level, the chosen phage capsid protein partner and the helper phage used in superinfection had a major impact. This was analysed first from the phage immunoassay data of the induction experiments (Figure 3A). The highest Fab/phage-ratio was obtained from hyperphage-packed Fab-p3 as anticipated. The next highest display level was observed in Fab-p3∆ followed by the VCS-M13-infected Fab-p3. The lowest Fab/phage-ratio was observed in the Fab-p9 library. The ranking according to the display level was unchanged by the induction condition, except at 1% glucose, at which, the display efficiency of the hyperphage-packed Fab-p3 library decreased significantly. Worthy of notice, the Fab signal measured from the phage bound on the anti-mouse IgG plate is a sum of two phenomena, both the number of fragments on a single phage particle as well as the share of the phage bearing at least one antibody molecule affect the immunoassay results. Their partial contribution in this assay system cannot be deduced.The display levels were determined next from the stocks to be used in panning experiments and compared to the results of the study conducted with the culture medium samples (Figure 3B). In the latter assay, the Fab display efficiency was normalized according to the infective titer instead of total phage mass. The primary phage stocks were produced in the presence of 100 μM IPTG. When the Fab/phage-ratio of the hyperphage-infected Fab-p3 sample was taken as 100%, the relative display levels of the other libraries were 22.7% (39.5 ± 13.4%), 4.9% (9.17 ± 8.5%) and 3.2% (2.4 ± 0.4%) in Fab-p3∆, VCS-M13 infected Fab-p3 and Fab-p9, respectively (display levels by the immunoassay given in parentheses). The display ranking was same as in the earlier assay, but there was a wider gap between the display efficiency of hyperphage-infected and VCS-M13-infected stocks, when normalized by infectivity.

The measured higher relative efficiency of hyperphage display by infectivity normalization is most probably due to the fact that the infective titer underestimates the total phage mass in hyperphage stocks [23]. Consequently, more hyperphage are added to the assay by infective titer than by total phage mass. In this study, the infectivity of the hyperphage stocks was restored by trypsination before infection experiments. However, trypsination increased the infective titer of hyperphage samples only threefold in our experiments, whereas others have reported even 100-fold increments depending on the fusion moiety [23]. Therefore, a full restoration of infectivity remains questionable. Trypsination did not have an effect on the infectivity of other phage stocks (data not shown).

The display efficiency of ScFvs in our phagemids has been evaluated earlier by protein L capture in another study [24]. The relative display efficiencies of four clones as ScFv-p3∆ fusions displayed with VCS-M13 infection were 1.2-10% compared to the hyperphage equivalents displayed in ScFv-p3 format. The display efficiency of the same clones as p9 fusions (in an amber-stop containing p9 vector produced from the suppressor strain XL-1) was < 0.01–0.06% compared to the hyperphage equivalents. Thus, the relative display efficiency of ScFv-p3∆ clones was lower and ScFv-p9 clones far lower than measured for Fab-p3Δ and Fab-p9 in this study. However, the ScFv and Fab assay results are not directly comparable due to the difference in the nature of the capture reagent (nor due to the amber stop), which was polyclonal in Fab assay and a monovalently interacting protein L in ScFv assay. If present, avidity effects due to the polyclonal nature of the capture molecules lead to slower off-rate in the Fab assay, which in turn leads to higher retention of phage and hence higher signal in the assay. This phenomenon would exactly increase the efficiency of monovalent display in relation to oligovalent display. The avidity effects of the polyclonal capture exerted on the hyperphage are minimal due to the intrinsic higher valency of the display format, which is eminent as a slow off-rate. At nanomolar (Kd) binding affinities, a change from mono- to bivalency prolongs the half-life of the capture-Fab-phage complex beyond the incubation times applied in the assay.

### Selections against streptavidin and digoxigenin

**Figure 4:**
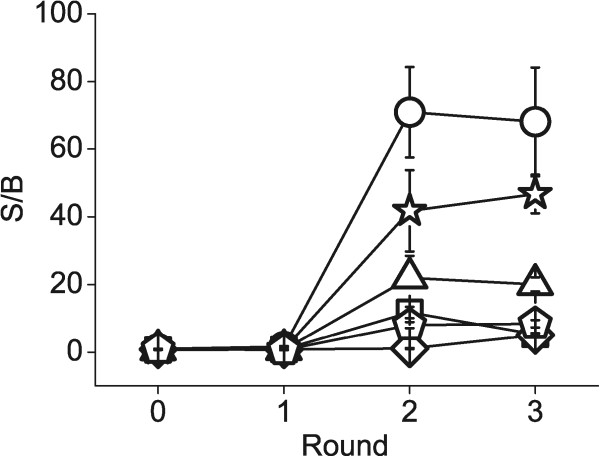
**Phage immunoassay of the first selection against streptavidin, rounds 0 – 3.** Circle: Hyperphage Fab-p3, square: VCS-M13 Fab-p3, pentagon: Hyper > VCS Fab-p3, triangle: Fab-p3Δ, star: ScFv-p3Δ and diamond: Fab-p9. 1 × 10^9^ rcfu phages were applied on STR and BSA wells and revealed by europium-labeled anti-phage antibody. The data is shown as the STR/BSA-signal ratio (signal-to-background, S/B) for each sample with the mean and standard deviation of three intra-assay replicas.

**Figure 5:**
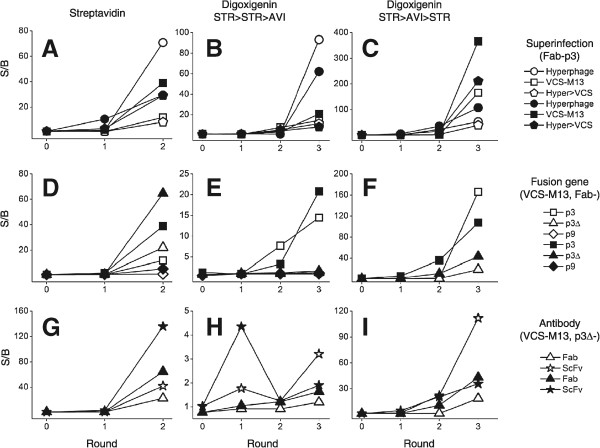
**Phage immunoassay of the enrichment against target antigens.** The enrichment of phages is shown in the three selection schemes: selection against streptavidin **(Panels A, D and G)**, selection against digoxigenin with STR>STR>AVI-carrier strategy **(Panels B, E and H)** and selection against digoxigenin with STR>AVI>STR-carrier strategy **(Panels C, F and I)**. Samples have been divided further according to superinfection **(Panels A, B and C)**, fusion gene **(Panels D, E and F)** and antibody format **(Panels G, H and I)**. Open symbols: first repeat and filled symbols: second repeat. 1 × 10^9^ rcfu phages were applied on STR and BSA wells (streptavidin) or DIG and biotin wells (digoxigenin) and revealed by europium-labeled anti-phage antibody. The data is shown as the STR/BSA- and DIG/biotin-signal ratio (S/B) for each sample. All sample points measured as triplicate (CV < 39% at all measured points with a median at 19%). Error bars excluded for clarity.

The libraries were selected twice against streptavidin coated on paramagnetic beads. A third round of selection did not improve enrichment further, except in Fab-p9 library (Figure 4) and therefore, further analysis of anti-STR selection was performed from rounds 0–2. The highest anti-STR signals on the second round stocks were obtained from hyperphage-infected Fab-p3 and VCS-M13-infected ScFv-p3∆ libraries in the first and second repeat, respectively (Figure 5A and G). A closer analysis of the anti-STR response in relation to the used helper phage was somewhat perplexing. In the first repeat, hyperphage was superior to VCS-M13 and hyper > VCS-strategy, but in the second repeat, the stocks demonstrated somewhat equal response (Figure 5A). The enrichment properties of the libraries were more predictable when grouping the libraries according to the fusion gene partner and antibody format. The efficiency of enrichment for the target-specific clones followed the general display levels of the libraries: p3∆ > p3 > p9 (Figure 5D) and, the S/B-ratios of anti-STR phage after the second round were two-fold higher in ScFvs than Fabs (Figure 5G).Performance of the libraries in more challenging panning conditions was carried out by selections against a model hapten digoxigenin (DIG, 391 g/mol). Digoxigenin was linked to biotin via a disulfide-bridge enabling elution by reduction (bio-SS-DIG). Two initial rounds of panning were conducted on streptavidin beads coated with bio-SS-DIG and the final round on avidin-coated microtiter plate. The ScFv-library was especially prone to the enrichment of antibodies against the STR-carrier in addition to digoxigenin, which can be seen as a decrease in the S/B-ratio (STR-wells without bio-SS-DIG as the background) of the 2nd round phage samples in phage immunoassay (Figure 5H). Due to this reason, the second round was repeated on avidin-wells coated with bio-SS-DIG. The final round in the latter panning was performed on streptavidin beads (STR > AVI > STR). As expected, higher S/B-ratio for digoxigenin was obtained from the STR > AVI > STR-passed phage than from STR > STR > AVI-passed phage (Figure 5B, E and H vs. C, F and I). Ranking of the anti-DIG phage according to the helper phage used was rather random, an observation also noted with streptavidin selections. One recurring observation was the faster enrichment of target-specific Fabs by p3 than p3∆ or p9 display in the DIG selections (Figure 5E and F).

### Single clone screening

**Figure 6:**
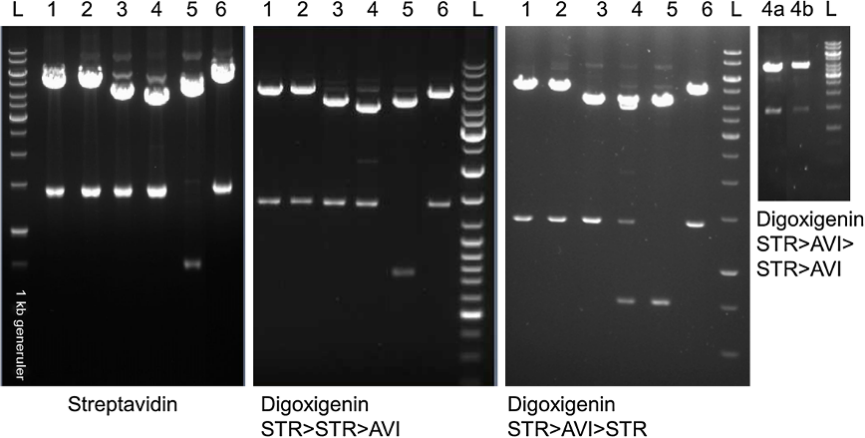
**SfiI-digests of phagemids after panning.** The DNA digests are from the second repeat of streptavidin (2nd round) and digoxigenin (3rd round) selections. Digestion patterns of the first repeats are identical. 1) Hyperphage/pEB30-Fab, 2) VCS-M13/pEB30-Fab, 3) VCS-M13/pEB32x-Fab, 4) VCS-M13/pEB92-Fab, 5) VCS-M13/pEB32x-ScFv, 6) Hyper > VCS/pEB30-Fab. The fourth DNA digest is from the repeated p9-Fab digoxigenin selection: 4a) VCS-M13/pEB92-Fab 3rd round and 4b) VCS-M13/pEB92-Fab 4th round.

The 2nd round anti-STR and 3rd round anti-DIG repertoires were cloned from phagemid to expression vector for screening. Samples of the SfiI-digests were monitored on gel to verify that the libraries had not contaminated during the panning and that there were no aberrations in the size of the antibody genes. In fact, a visible amount of pEB32x-ScFv DNA was found on the pEB92-Fab lanes in the final anti-DIG STR > AVI > STR-selections (Figure 6), and therefore, the SfiI-fragments corresponding to the size of the Fab fragments were gel extracted from Fab-p9 library lanes for ligation. No contaminations were observed in other samples, which were then cloned with a gel extraction free method from the chloramphenicol-resistant donor phagemid to the ampicillin-resistant destination vectors. Due to the contamination, the phage immunoassay results of Fab-p9 libraries from STR > AVI > STR-selection are not shown in Figure 5, as the ScFv-repertoire clearly had a major impact on the assay results. Fab-p9 libraries were also at the highest risk for contamination by other libraries in the study set as the target-specific enrichment of clones in the Fab-p9 library was the poorest.

Anti-STR and anti-DIG antibodies from STR > STR > AVI-selection were screened as alkaline phosphatase fusions and anti-DIG antibodies from STR > AVI > STR-selection as constant light (CL) IgG domain fusions. The use of mouse CL domain as the fusion partner enabled direct higher stringency screening with Eu-labeled digoxigenin than achieved with the indirect detection via alkaline phosphatase. 42–52 clones were screened and four clones with the highest S/B-ratio were taken for more detailed studies from each output.

**Figure 7:**
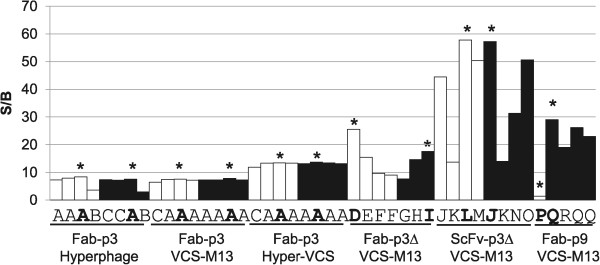
**Affinity ranking STR-binders with 5 nM europium-labeled STR.** Genetical identity of each clone revealed by capital letters. Star: highest S/B in the group of four, white bars: first repeat and black bars: second repeat. The assay was performed twice with similar results.

After two rounds of panning against streptavidin, over 50% of all screened clones bound the target, except in the Fab-p9 repertoires, and over 90% hit rates were observed in VCS-M13-infected ScFv-p3∆ and Fab-p3 sample repertoires (Table 2). All clones that passed a secondary screening with 5 nM STR-Eu were sequenced. Only three unique clones were found among the Fab-p3 members (Figure 7). Two of them were present in all Fab-p3 libraries irrespective of the used helper phage, and the more abundant clone was clearly dominating the outputs with 4/8, 7/8 and 7/8 shares of the sequenced clones originating from the hyperphage, VCS-M13 and hyper > VCS selections, respectively. Higher clonal diversity was obtained from p3∆ displayed Fab and ScFv libraries with 6/8 different clones in both. Although the enrichment of target-specific clones in the Fab-p9 library was poor and only five clones were taken further from the primary screening, 3/5 different clones were encountered in the output.

**Table 2:** **Primary and secondary screening of antibodies against streptavidin and digoxigenin**

		Streptavidin					Digoxigenin STR > STR > AVI					Digoxigenin STR > AVI > STR					
		Primary screening			Secondary Scr.		Primary screening			Secondary Scr.		Primary screening				Secondary Scr.	
Library	Exp.	Screened	Hits	%	Screened	Hits	Screened	Hits	%	Screened	Hits	Screened	Hits	%	S/B >20	Screened	Hits
Hyperphage	I	46	35	76%	4	4	42	37	88%	4	4	45	31	69%	8 (26%)	4	3
pEB30-Fab	II	44	25	57%	4	4	42	38	90%	4	4	45	20	44%	5 (25%)	4	4
VCS-M13	I	47	41	87%	4	4	42	37	88%	4	4	45	37	82%	9 (24%)	4	4
pEB30-Fab	II	35	33	94%	4	4	42	40	95%	4	4	45	39	87%	22 (56%)	4	4
Hyper/VCS	I	46	38	83%	4	4	42	41	98%	4	4	45	36	80%	7 (19%)	4	4
pEB30-Fab	II	47	38	81%	4	4	42	38	90%	4	4	45	37	82%	7 (19%)	4	4
VCS-M13	I	43	32	74%	4	4	42	3	7%	4	2	45	3	7%	2 (67%)	3	3
pEB32x-Fab	II	43	35	81%	4	3	42	8	19%	3	3	45	5	11%	0 (0%)	4	4
VCS-M13	I	44	41	93%	4	4	42	26	62%	3	3	45	21	47%	15 (71%)	4	4
pEB32x-ScFv	II	52	47	90%	4	4	42	26	62%	4	4	45	17	38%	11 (65%)	4	4
VCS-M13	I	47	1	2%	1	1	42	0	0%	0	0	45	0	0%	0 (0%)	N.D.	N.D.
pEB92-Fab	II	43	17	40%	4	4	42	3	7%	3	1	45	0	0%	0 (0%)	N.D.	N.D.
STR > AVI > STR	IIIa											45	0	0%	0 (0%)	N.D.	N.D.
STR > AVI > STR > AVI	IIIb											45	4	9%	N.D.	4	3

**Figure 8:**
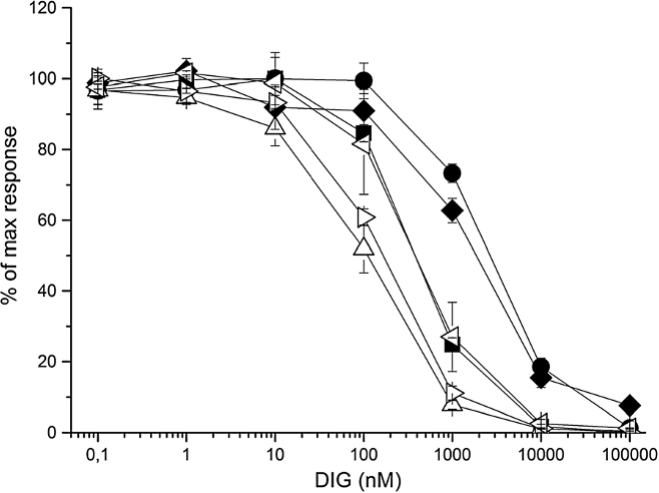
**IC50 assay of the three highest affinity anti-DIG antibodies from Fab (filled symbols) and ScFv (open symbols) repertoires.** Clone “filled square” found in Fab-p3, Fab-p3∆ and Fab-p9 repertoires. Clone “filled circle” found in Fab-p3 and clone “filled diamond” in Fab-p3∆ repertoire. Open triangles: from ScFv-p3∆ repertoire. The plots represent the mean of three independent measurements with standard deviation.

In the anti-DIG screening from the STR > STR > AVI-selection, over 80% hit rates were obtained for digoxigenin in all Fab-p3 libraries (Table 2). Fab- and ScFv-p3∆ libraries suffered from high frequency of streptavidin binders that was already foreseen by phage immunoassay. Consequently, the frequencies of anti-DIG antibodies were 19% and 7% for the first and second repeat of Fab-p3∆ library selection, respectively, and 62% for both ScFv-p3∆ library selections. The lowest frequency of anti-DIG clones was again obtained by Fab-p9 display. Only 3/42 clones were considered to be positive in the first output of the anti-STR p9-Fab library selection and none in the second output. Like earlier, four clones with the highest S/B of each output were taken for a secondary screening, which was performed as IC50 assay. Altogether four clones failed to respond in the IC50 assay, of which, two originated from Fab-p9 and the remaining from Fab-p3∆ library. Sequencing of the clones that were confirmed to be reactive against DIG revealed that a single clone dominated all Fab-p3 derived libraries (Table 3). The same clone was also found as a single representative among Fab-p9 and Fab-p3∆ members. It was also the highest affinity anti-DIG Fab in the set with IC50 value 442 ± 37 nM for DIG (Figure 8).

**Table 3:** **Sequenced anti-DIG clone diversity and highest observed affinities**

		Digoxigenin STR > STR > AVI			Digoxigenin STR > AVI > STR		
Library	Helper phage	N	D	Highest aff. (IC50)	N	D	Highest aff. (IC50)
Fab-p3	Hyperphage	8	1	442 ± 37 nM	8	4	442 ± 37 nM
Fab-p3	VCS-M13	8	1	442 ± 37 nM	8	2	442 ± 37 nM^a^
Fab-p3	Hyper > VCS	8	1	442 ± 37 nM	8	1	442 ± 37 nM
Fab-p3Δ	VCS-M13	7	4	442 ± 37 nM	7^b^	5	1.5 ± 0.1 μM
ScFv-p3Δ	VCS-M13	8	4	158 ± 20 nM	8	6	112 ± 24 nM
Fab-p9	VCS-M13	3	2	442 ± 37 nM	0	0	N.D.

N: secondary screened and sequenced clones. D (diversity): number of unique clones.

^a^The other output dominated by a clone with IC50 value 2.7 ± 0.4 μM.

^b^One read missing due to bad quality template.

Screening the STR > AVI > STR-selection with 65 nM DIG-Eu resulted in slightly lower overall hit rate compared to the earlier alkaline phosphatase screening (Table 2). The highest hit rates were obtained from libraries originating from the monovalent Fab-p3 and the lowest hit rates from Fab-p9 libraries. Actually, none of the picked clones from Fab-p9 repertoire showed adequate reactivity towards digoxigenin to exceed the set cut-off. Taking into consideration only the share of hits with S/B > 20, the best performing repertoire was the ScFv library with 2/3 of the clones belonging to this subclass (Table 2). The highest clonal diversity was found in ScFv-p3∆ and Fab-p3∆ libraries with 6/7 and 6/8 unique clones. In good accordance with the screening results and the number of unique clones, the highest affinity anti-DIG clone was a ScFv with an IC50 value 112 ± 24 nM for DIG. The same clone, that came up from the STR > STR > AVI-selection, was present also in the STR > AVI > STR-selection outputs, but only in the Fab-p3 repertoires. In addition, four unforeseen anti-DIG Fab clones were obtained from the Fab-p3 libraries and another four from Fab-p3∆ libraries. However, the biggest contribution to unique members was achieved by sampling the ScFv repertoire as the clones from the latter STR > AVI > STR-screen did not have any common member with the earlier STR > STR > AVI-screen.

Due to the contamination of the p9-Fab repertoire with the ScFv-p3Δ clones during the selection, the p9-Fab repertoire was refreshed from the glycerol stocks and selected again against digoxigenin for four rounds of biopanning with alternating STR > AVI > STR > AVI-carrier sequence. All selections were performed against solid-phase-coated bio-SS-DIG to maximize the number of binding clones. After four rounds of panning, a sixfold difference in the number of output colonies was observed from the antigen selection compared to the background selection. No contamination was observed in the extracted phagemid DNA after 3rd or 4th round of selection by gel electrophoresis (Figure 6). The phagemid DNA was digested with SfiI and the Fabs cloned as fusions with the alkaline phosphatase gene for the indirect ELISA screening. No hits were found from the 3rd round output by screening 45 clones (Table 2, IIIa). After four rounds of selection 4/45 positive clones were identified, of which 3/4 were confirmed to bind digoxigenin in the secondary screening (Table 2, IIIb). Sequencing the three clones revealed a single novel genotype.

### Ranking the libraries

Because the number of unique specific clones was low and selections were carried out on only two different antigens, it is naturally not possible to give an explicit answer to the question, which of the different display formats is the best tool for accessing the largest number of unique specific antibody clones in the library. But clear trends were observed in the number of obtained specific clones, which followed almost identical pattern in STR and DIG selections. ScFv-p3∆ library yielded altogether 10 unique clones against DIG followed by Fab-p3∆, hyperphage-packed Fab-p3, VCS-M13-packed Fab-p3 and Fab-p9 with 8, 5, 2 and 2 unique clones, respectively. At least a single copy of the same clone, which turned out to be the highest affinity anti-DIG Fab in the study, was retrieved in 13/15 analysed Fab library outputs. The affinities of the other anti-DIG Fab clones were at least three times lower for digoxigenin with IC50 values residing in the range 1 – 10 μM. In contrast, several clones with similar IC50 values between 100 nM and 1 μM were obtained from the ScFv-p3∆ repertoire (Figure 8).

It is not possible to apply similar affinity ranking for anti-STR clones. The interactions of the tetravalent antigen with the bivalent alkaline phosphatase antibody fusion are inherently multivalent and the apparent affinities are dependent on the locality of the epitopes. The clonal diversity analysis of the outputs supports the findings from the DIG selections as 6, 6, 3, 2 and 3 unique clones were obtained from ScFv-p3∆, Fab-p3∆, hyperphage-packed Fab-p3, VCS-M13-packed Fab-p3 and Fab-p9 library, respectively. The combination of hyperphage packing on the first round for oligovalent display followed by VCS-M13 packing on the following rounds for monovalent display did not provide access to any unforeseen clones in any of the experiments. This may however be due to the loss of functional diversity connected to the Fab-p3 display, which reduced the number of unique clones to a level, where valency modulation cannot make a difference.

In the light of this study, ScFv is an attractive display format, but it is rarely the favoured antibody fragment for applications. There are examples in which conversion from ScFv to Fab causes affinity decrease or even specificity loss [25], although in some cases the reason may be an unnoticed change in valency as ScFv may form dimers or higher multimer structures [26], whereas Fab is considered strictly monomeric molecule. Also conversion of ScFv to full-length IgG is not as straightforward as conversion of a Fab molecule. On the other hand, if large diversity and fast generation of initial binding reagents is required, library construction on the smaller ScFv-gene is easier than on Fab. Also the observed affinities of the retrieved ScFv clones against DIG were superior to the Fab clones. Direct comparison of affinities is indeed possible by the IC50 assays, in which the analyte and tracer are naturally monomeric ruling out the possibility of avidity effects.

### On the p9 display

The enrichment of target-specific clones in p9 libraries was inferior to p3 and p3Δ libraries in our study. In contrast to this, there are reports in which the performance of p9 displayed ScFv [27] or Fab [28, 29] libraries have been esteemed to be equal or even superior to p3 display. However, a closer look at these references reveals that valid side-by-side comparison between p3 and p9 were not carried out. In the ScFv-p9 study the performance of the library was experienced to be superior to the earlier version displayed as p3 fusions [27]. This may, however, be also due to the fact that the newer naïve PelB-ScFv-p9 library was constructed from a larger source repertoire and by a more efficient method than the earlier p3 library [27]. In the other studies on Fab-p9 display, conclusions were withdrawn by meta-analysis. P9 display was concluded to be a comparable platform to p3 display, because similar affinities and numbers of unique clones were obtained as reported by others using p3 display [28, 29]. We have also shown earlier that ScFv-p9 display truly is a working concept [24], but as the enrichment of binding clones also in ScFv-format seemed to be faster by p3Δ display, our latest ScFv libraries are displayed as p3Δ fusions [14].

There is a myc-tag and flexible linker between Fab and p9, which are lacking from the p3 constructs. Naturally, it cannot be excluded that the sequence of the linker has an effect on the selection properties of the p9 repertoire. However, in the other p9 library initiatives the fusion gene constructions have been similar, such as PelB-ScFv-Flag-G_4_S_1_-p9 [27], PelB-Fd-TSG_4_S_1_-p9 [29] and PelB-ScFv-AAAGSKDIR-p9 [30]. Myc-tag is also used in p3 display libraries without reported effect on the display efficiency [31]. Therefore, we consider the presence of myc-tag an unlikely explanation for the observed decreased display and selection performance in p9 experiments. This is further supported by the study by Løset et al., in which, a tenfold display efficiency difference between p3 and p9 in favour of p3 was observed [30].

The slow enrichment of binding clones in p9 due to lower display efficiency is analogous to the findings of a study in which, the selection performance of a naïve ScFv library was monitored before and after pre-selection with protein L for correctly folded clones [32]. The enrichment of the target binding clones was more efficient in the pre-selected library that contained a higher initial share of displayed moieties. Moreover, parallel selections against a more challenging target resulted in the retrieval of specific clones only from the functionally pre-selected repertoire [32], similar to our observation in the latter DIG selections with the p9 library. However, in contrast to display on p3 or p3Δ, p9 display may indeed be a superior choice for affinity maturation experiments due to lower display valency [24]. The favourable characteristic of p9 display was also observed by Shi et al. as the copy number of retrieved binders correlated positively with improved affinity [28]. This correlation is absent in libraries containing mixed valency of display moieties by default.

Shi et al. made a surprising observation regarding the framework combinations of VL and VH pairs [28]. Despite the equal provision of twelve framework combinations (three VH domains and four VK domains) in Fab format for selections, the germline IGKV3-20 (also known as A27) gene was heavily underpresented among the obtained antigen specific clones [28]. The scaffold in the present study is a pair of germline IGHV3-23 and IGKV3-20 genes and this may be the reason for the slow enrichment. It remains to be elucidated, however, is the poor performance really scaffold-dependent, and if so, what is the mechanism of display censoring. A notable observation in the study by Shi et al. was also, that IGKV3-20/IGHV3-23 and IGKV3-20/IGHV1-69 -pairs were most efficiently displayed on phage [28], which is, prima facie, in contradiction to the censoring hypothesis.

Løset et al. have made an interesting observation on the role of PelB leader peptide in p9 display [30]. According to them, in PelB-directed p9 display, helper phage genome instead of phagemid genome, is predominantly packed into the forming virions, which decreases the number of correct geno-phenotype units in selections [30]. This phenomenon was reversed by removing the N-terminal signal sequence, which resulted in superior enrichment of the target-specific clones in the p9 library as compared to conventional p3 display [30]. However, the removal of the signal peptide resulted in a tenfold reduction in display efficiency by phage immunoassay [30], indicating that the elimination of helper phage was the major factor for the observed improvement in selections. Also the present study supports this observation as there was disparity in the measured Fab display per phage ratios as measured by total phage mass (2.4 ± 0.4%) and infectivity (3.2%, infected cells counted as cmR colonies), which may be explained by the presence of VCS-M13 in the phage stock.

### Critical review of the experimental set-up

Comparison of phage display formats in library scale is a demanding challenge in terms of the amount of work required and righteous study design, and several technical compromises have been undertaken, which are discussed here. The comparison was conducted from a practical point of view and therefore, the primary phages for panning were produced from equal volume of cell culture from all libraries. The seeding number of cells was in the range of 10^10^ cfu, which was tenfold more than the initial number of transformants from Fab-p3∆, but only twofold more than the number of transformants in Fab-p9 and ScFv-p3∆ libraries. Another way of comparison would have been to take an equal overpresentation of seeding cells to the number of transformants, which would have lead to different cell culture volumes, but this was not undertaken.

Another point, which most probably had an effect on the selection outcome, was the number of input phage for the first round. We wanted to avoid manual handling and therefore, performed the selections on Kingfisher magnetic bead processor. Our instrument had a limited reaction volume of 200 μl. Therefore, 2 × 10^12^ cfu input phage were taken from each library for the first round, which corresponds to the same phage concentration as 10^13^ cfu in a more typical 1 ml binding reaction. With the following assumptions we can visualize the possible error that arises from the limited input.

Firstly, a simplification is made that the display efficiency mirrors merely the number of antibody displaying phage and the number of Fab displaying phage are rounded up from the results of this study to be 100%, 20% and 2% for hyperphage-packed Fab-p3, VCS-M13-packed Fab-p3∆ and Fab-p9, respectively. Secondly, the true number of unique clones is fixed to 10^9^ in all repertoires, which in reality is probably less than that due to the PCR methods employed in Fab cassette assembly prior to final ligation. Then, every unique antibody in Fab-p3∆ repertoire would be physically displayed as 400 copies ((20% × 2 × 10^12^)/10^9^), but in Fab-p9 repertoire only as 40 copies ((2% × 2 × 10^12^)/10^9^). Due to the 20-fold lower titer of the hyperphage stock compared to VCS-M13, the input was also 20-fold less on the initial round, which translates into ((100% × 1 × 10^11^/10^9^) 100 copies per clone.

In a typical phage display experiment, a 100-fold over presentation of each clone is recommended [33], and this criterion is fulfilled in the current study with all other libraries, except Fab-p9. Another way to normalize at this stage, would be to take different amount of phage particles for the initial round of panning to take into account the differences in display efficiency aiming at an equal copy number of each library clone. This was however not undertaken due to the inaccuracies involved in translating the relative display efficiencies correctly into numbers of antibody fragment displaying phage particles.

## Conclusions

We were able to display the same Fab library as a fusion to all tested capsid proteins of filamentous phage. There was a decrease in display efficiency of the Fab molecules with a sequence: hyperphage-packed Fab-p3 > VCS-M13-packed Fab-p3∆ > VCS-M13-packed Fab-p3 > VCS-M13-packed Fab-p9. The poor display of Fab on p9 was reflected as markedly slower enrichment of binding clones or even as a failed experiment in the applied selection conditions. Selecting for more than three rounds is therefore recommended with a similar construction as Fab-p9 as exemplified by the last panning experiment with p9, in which specific Fab clones were obtained only after four rounds of selection. There was a significant loss of functional diversity in Fab-p3 library after a single pass of phage packing, irrespective of the helper phage used. The enrichment of frameshift mutants was also observed with ScFv-p3∆, but to a lesser extent. The loss of functional diversity in phage packed Fab-p3 library was further evidenced by the low number of retrieved unique specific Fab clones, especially from VCS-M13-superinfected selections. As highlighted by these observations, a better overview of the true functional diversity of a library is obtained by sequencing the library after phage packing. Currently, even the most precisely analysed antibody repertoires have been sequenced before phage packing [34, 35], which is not the same as the actual repertoire used for the selections, which could be analysed post phage packing.

The oligovalently displayed Fab-p3 library retained more different clones through the selections than the monovalent display (8 vs. 4 unique clones for STR and DIG), although the purely oligovalent or hyper > VCS-strategy could not provide any higher affinity clone than was already obtained by purely monovalent display. Overall, the highest numbers of unique specific clones were obtained with Fab- and ScFv-p3Δ display and, the highest affinity anti-digoxigenin antibody fragments in this study were obtained from the ScFv-repertoire with IC50 values 112 ± 24 nM and 158 ± 20 nM for digoxigenin. A fast enrichment is not always a guarantee for quality. This was observed as the expansion of the most widespread anti-DIG monoclone on the third round of digoxigenin panning, especially in the monovalent Fab-p3 library. As a conclusion, the results at hand underline the importance of considering the display related aspects, to avoid wrong conclusions of the library potential, as the chosen display format may influence the outcome of the experiment.

## Methods

### Vectors, enzymes and kits

The construction of phagemid pEB30 for full-length p3 display is described in Huovinen et al. [13]. The construction of phagemid pEB32x for truncated p3 display, and screening vectors pLK01 and pLK06 are described in Huovinen et al. [14]. Phagemid pEB92 was constructed by replacing the gene for truncated p3 in vector pAK200 [22] with p9 amplified from the helper phage VCS-M13 (Agilent Technologies, Santa Clara, CA). Myc tag and Gly_2_SerGly_2_ linker preceding the p9 gene were introduced by PCR primers. Tet-cassette-p9 was cloned forward into pEB30 with BamHI and HindIII sites replacing the full-length p3 gene. All restriction enzymes in this work were purchased either from New England Biolabs (Ipswich, UK) or from Thermo Scientific (Waltham, MA, USA). Ligations were carried out with T4 DNA ligase and PCR either with Pfu DNA polymerase or Phusion DNA polymerase, which were all purchased from Thermo Scientific (Waltham, MA, USA). Purification of PCR products and gel extraction were performed with kits from Qiagen (Hamburg, Germany), whereas minipreps were prepared with a kit from Thermo Scientific (Waltham, MA, USA).

### Library construction

The diversification of the VL and VH domain genes and the purification of the light (VL-CL-BLA + VH-CH1) and heavy (VL-CL + VH-CH1-BLA) chain repertoires as beta-lactamase fusions is same as described for ScFvM library assembly in [14]. For the final Fab assembly, light chain repertoire was amplified with primers WO375 and TH28 and heavy chain with primers TH27 and pAK400rev using Phusion DNA polymerase and beta-lactamase-selected purified DNA as the template. TH27 and TH28 primers create BspQI restriction sites at the intervening sequence region between light and heavy chains. After PCR purification with QIAquick PCR Purification Kit, the amplified repertoires were digested with BspQI and ligated with each other in equimolar mixture. The primers used in this study are listed in Table 4.

**Table 4:** **Primers used in the study**

Primer	Sequence
WO375	5′-TCACACAGGAAACAGCTATGAC-3′
TH27	5′-ACCATTGCTAGCTCTTCTTAATCTAGATTAATTAAAGGAGAATTGAATG-3′
TH28	5′-ACCATTGCTAGCTCTTCTTTAACACTCTCCCCTGTTGAAGC-3′
pAK400rev	5′-CGCCATTTTTCACTTCACAG-3′
ULa08_10	5′-GTGCACCCAACTGATCTTC-3′

The ligations were purified with PCR Purification Kit, amplified with primers WO375 and Ula08_10 using Pfu DNA polymerase and purified again as above. The PCR products were digested with SfiI and purified with PCR purification kit. The completeness of digestion was confirmed by sample analysis on 1% agarose gel. 100 μg of vectors pEB30, pEB32x and pEB92 were digested with SfiI and gel-purified. SfiI-digestions were supplemented with restriction enzyme EcoO109I to linearize supercoiled vector DNA at the TetR-cassette, which is subsequently removed in vector extraction. Linearization of the vector with EcoO109I yielded better separation of the linear SfiI-vector fragment from the undigested supercoiled DNA.

2-3x molar excess of diversified SfiI-digested Fab fragments were ligated to 16 μg digested vector as eight sublibraries, each equivalent to a different CDR-H3 loop length. Ligations were purified with PCR purification kit, eluted into 30 μl 5 mM Tris-Cl pH 8.5 and 12 μl of each ligation was mixed with 420 μl fresh SS320 electrocompetent cells. The cells were divided in 6 electroporation cuvettes (0.1 cm gap width, Bio-Rad, Hercules, CA, USA) 70 μl/cuvette and electroporated with settings 200 Ω, 25 μF, 1.25 kV (Gene Pulser II, Bio-Rad, Hercules, CA, USA). The cells were suspended in 2 × 1 ml SOC prewarmed at 37°C. The recovery suspensions of six similar transformations were combined in a 100 ml erlenmeyer flask containing 12 ml prewarmed SB and incubated at 37°C with 200 rpm shaking for 1 h. 100 μl samples from 1/1000 and 1/10000 dilutions were plated on LA containing 0.5% glucose, 10 μg/ml tet and 25 μg/ml cm in triplicate from separate dilution series. The rest of the cells were plated on two 24 × 24 cm bioassay dish (WVR, Radnor, PA, USA), ~12 ml/ plate. Large plates were incubated at 30°C and small plates at 37°C o/n. The library size was counted from the small dilution plates. Minipreps were prepared from the colonies for sequencing. All sequencing in this study was performed either by the Sequencing Service unit of Center of Biotechnology (Turku, Finland) or by Macrogen Inc. (Seoul, Korea). Cells were collected from each large plate with 15 ml SB, glycerol was added to final 16% concentration and the cells were stored at -70°C for phage production.

### Display induction experiments

Equal number of cells from each eight sublibraries was combined for display induction experiments. The cells were diluted to OD600nm 0.05 in SB medium containing 25 μg/ml cm, 10 μg/ml tet and 1% glc and grown to OD600nm 0.3 at 37°C with 300 rpm shaking. Superinfection was carried out at 10× and 20× multiplicity of infection with hyperphage (PROGEN, Heidelberg, Germany) and VCS-M13 (Stratagene/Agilent Technologies, Santa Clara, CA, USA) respectively, for 30 min at 37°C. Infected cells were divided into 4 ml aliquots in 14 ml round-bottom culture tubes and centrifuged with Eppendorf Centrifuge 5810 R (Hamburg, Germany) for 5 min at 4000 rpm. The supernatant was removed and pellet resuspended in 4 ml SB supplemented with 25 μg/ml cm, 10 μg/ml tet, 5 mM MgCl_2_ and (1) 1% glc, (2) 0.5% glc with 500 μM IPTG, (3) 0.5% glc, (4) 0.2% glc, (5) 0.1% glc, (6) 0.05% glc, (7) 0.025% glc, (9) 10 μM IPTG, (10) 100 μM IPTG, (11) 250 μM IPTG or (8) nothing. The cultures were incubated at 26°C with 300 rpm shaking for 1 h, 30 μg/ml kan was added and incubation continued o/n. Cells were centrifuged at 10 000 rpm with Sorvall Evolution RC (Thermo Scientific, Waltham, MA, USA) for 10 min and the phage-containing supernatant was used for assays.

Phage supernatant was diluted 1/10, 1/100 and 1/1000 in Kaivogen assay buffer (Kaivogen, Turku, Finland) and 100 μl samples were applied in triplicate on 96-well Maxisorp plate (Nunc Int., Roskilde, Denmark) coated with rabbit anti-mouse IgG (RAM) (Sigma-Aldrich, St. Louis, MO, USA), and streptavidin plate (Kaivogen, Turku, Finland) coated with 100 ng/well biotinylated mouse anti-M13 antibody (Dept. of Biochemistry, University of Turku, Finland). Phage were incubated with low shaking for 1 h, washed four times and detected with 130 ng/ml europium-labeled rabbit anti-fd (anti-phage) Ab (Sigma-Aldrich, St. Louis, MO, USA). The label was incubated for 1 h at RT with slow shaking and washed four times. Delfia Enhancement solution (Perkin-Elmer, Turku, Finland) was added and incubated further 15 min. Time-resolved fluorescence signal was measured with Victor 1420 Multilabel Counter (Wallac, Turku, Finland).

### Determination of display efficiency

Display efficiency was determined first from display induction data by choosing dilutions of the samples at which both anti-Fab and anti-phage response were in the linear detection range (500 – 25 000 CPS). The counts were multiplied with the corresponding dilution factors and the obtained total Fab-signal was divided by the total phage-signal to obtain Fab/phage-ratios. The Fab/phage-ratios were divided by the highest observed Fab/phage-value of hyperphage-infected pEB30-Fab library for plotting relative differences. Display efficiencies of the final display phage were determined using RAM plate assays and infective titers in an analogous manner.

### Library phage production and selections

Equal number of cells of all sublibraries were combined and inoculated into 500 ml SB containing 25 μg/ml cm, 10 μg/ml tet and 1% glc to OD600nm 0.1. Cells were grown at 37°C with 250 rpm until OD600nm 0.3 - 0.5, infected with helper phage as in induction experiments and collected by centrifugation with Sorvall Evolution RC (Thermo Scientific, Waltham, MA, USA) for 10 min with 5000 rpm. Cells were resuspended in 500 ml SB containing 25 μg/ml cm, 10 μg/ml tet and 5 mM MgCl_2_ and incubated for 1 h at 30°C with 250 rpm. The cultures were supplemented with 100 μM IPTG and 30 μg/ml kanamycin and incubation continued at 26°C with shaking at 250 rpm o/n. Phage stocks were prepared with PEG/NaCl-precipitation according to standard protocol. Phage titers were determined by infecting mid-log XL-1 Blue cells with a series of diluted samples and plating 100 μl samples on LA containing 25 μg/ml cm, 10 μg/ml tet and 0.5% glc. Colonies were counted after o/n incubation at 37°C. Minipreps were prepared from colonies on dilution plates for sequencing the repertoires after single phage passage.

Selections against STR and DIG were performed as described in [14] for “parallel selections” with Kingfisher magnetic bead processor (Thermo Scientific, Waltham, MA, USA) using STR-coated M-280 Dynabeads (Invitrogen Dynal, Oslo, Norway). Panning of anti-DIG antibodies on avidin carrier was performed in microtiter wells. The number of input phage on each round is listed in Table 5. Due to lower titers of the hyperphage-superinfected stocks, from the hyperphage-produced stocks 20 times less particles were applied for the first and second round of panning against STR and DIG than from the VCS-M13-produced stocks. The third round of selection against STR was performed with 50 μg STR beads with six washes. In addition, the third round anti-STR phage were exposed to 5.6 μM STR for further 2 h (50 times more than on beads), after which, beads were collected and transferred forward for elution. Phage from digoxigenin selections were eluted with 50 mM DTT on the first and 10 mM DTT on the following rounds.

**Table 5:** **Number of input phage for selections**

	STR		DIG SSA		DIG SAS	
Round	VCS-M13	Hyperphage	VCS-M13	Hyperphage	VCS-M13	Hyperphage
1	2 × 10^12^	1 × 10^11^	2 × 10^12^	1 × 10^11^	2 × 10^12^	1 × 10^11^
2	2 × 10^11^	1 × 10^10^	2 × 10^11^	1 × 10^10^	2 × 10^11^	1 × 10^10^
3	2 × 10^11^	1 × 10^10^	2 × 10^10^	1 × 10^10^	1 × 10^9^	1 × 10^9^

### Phage immunoassay

For monitoring enrichment of anti-STR clones, 1×10^9^ relative colony forming units (rcfu) of phages, as measured by phage immunoassay, were added from each round on STR and BSA wells as triplicate, incubated for 1 h with low shaking, washed four times and detected with 25 ng/well Eu-labeled mouse anti-phage antibody (Dept. of Biochemistry, University of Turku, Finland) as described above. For anti-DIG phage immunoassay, STR-strips (Kaivogen, Turku, Finland) were coated for 30 min with a saturating amount of bio-SS-DIG and biotin and washed four times. 1×10^9^ rcfu phage per well from different panning rounds were added on both strips in triplicate and the assay was continued as above.

### Primary clone screening

The infected output cells from the second (STR) and third (DIG) round of panning were collected from Ø 10 cm LA after o/n incubation at 30°C. DNA was extracted from half of the output cells with a miniprep kit and the inserts cloned into screening vectors pLK06 and pLK01 as described in [14]. Colonies were picked with sterile tooth picks on round bottom 96-Well plate (Sarstedt, Newton, NC, USA), expressed and lysed with a combination of freezing and lysozyme treatment in 200 μl volume as also described in [14]. The lysate was centrifuged at 4000 × g for 30 min and the supernatant used for primary screening assays.

Expression supernatant was diluted 1/10 in Kaivogen (Turku, Finland) assay buffer for screening. For STR screening, 200 μl diluted expression supernatant was added on STR- and BSA-coated strips, incubated for 1 h at RT with low shaking and washed four times. Bound clones were revealed by adding 100 μl alkaline phosphatase substrate buffer per well containing 1 mM pNpp (Sigma, St. Louis, MO, USA) and 10 mM MgCl_2_ in TBS pH 7.5. After 2 h incubation with low shaking assay plates were measured with 1420 Victor multilabel counter (Wallac, Turku, Finland) at 405 nm. Anti-DIG clones from the STR >10 STR > AVI-selection were screened with the same protocol. For DIG screening, STR wells were coated to saturation with bio-SS-DIG and biotin for the antigen and background signals, respectively. The expression supernatants of the clones from the anti-DIG STR > AVI > STR-selection (Fab/ScFv-CL) were diluted 1/5 in Kaivogen (Turku, Finland) assay buffer and applied on RAM plates 100 μl per well. RAM plates were incubated for 2 h at RT with low shaking, washed four times and the bound clones revealed with 100 μl 65 nM DIG-Eu. Labeled antigen was incubated for 2 h with low shaking at RT, washed four times and europium measured as in display induction assays.

The primary screening data for STR binders was analysed by dividing the signals obtained from the STR wells with the signals obtained from the BSA-blocked wells for the same samples. The primary screening data for DIG binders from the STR > STR > DIG-selection, in which the binders were analysed as alkaline phosphatase fusions, was analysed in a similar fashion by dividing the signals from the DIG wells with the signals obtained from the biotin wells for the same samples. The primary screening data from the anti-DIG STR > AVI > STR-selection was analysed by dividing the signal obtained from the europium-labeled DIG with the mean of the signal from the six background wells (SB medium only in the inoculation phase) present on each plate.

### Secondary clone screening and IC50 assays

For sequencing and secondary assay, the selected primary clones were streaked on LA containing 100 μg/ml amp and 1% glc and grown o/n at 37°C. Single colonies were picked, grown at 37°C in 5 ml SB supplemented with 100 μg/ml amp and 1% glc for o/n. The clones were re-expressed in 5 ml cultures as described in primary screening. For anti-STR secondary screening, freshly produced lysates were diluted 1/5 in Kaivogen assay buffer and 100 μl added per well in triplicate on goat anti-rabbit (GAR) IgG plate, which was precoated with polyclonal rabbit anti-bacterial alkaline phosphatase IgG (7.5 mg/ml, LifeSpan Biosciences, Seattle, WA, USA) (GAR-Anti-AP). The assay was incubated for 1 h and washed four times. Then, 100 μl 5 nM STR-Eu was added, incubated for 1 h, washed four times and the signal measured as in display induction assays.

For IC50 assays of anti-DIG clones, AP-fusions were coated on GAR-anti-AP plates and CL-fusions on RAM plates as described above. A dilution series of free DIG from 100 μM to 10 pM (Sigma, St. Louis, MO, USA) was prepared in Kaivogen assay buffer supplemented with a final concentration of 12.5 nM Eu-DIG. The mix was applied on the anti-DIG clones, 200 μl per well, and incubated for 2 h with low shaking at RT. The plate was washed four times and europium measured as above. Final IC50-values for digoxigenin was measured similarly with three replica wells for each dilution in at least three independent experiments.
